# Supplementary material for: Baseline Characterization of the Gut Microbiota of Field and Colony Populations of Phlebotomus tobbi and Preliminary Assessment of the Anti-Leishmanial Activity of Cultivable Bacteria
Source: Pathogens. 2026 Jun 23;15(7):658. doi: 10.3390/pathogens15070658 (PMC13416292; doi:10.3390/pathogens15070658)
Supplement: Supplementary file 1 [file pathogens-15-00658-s001.zip › pathogens-4357168-File S1/Taxa2ASV_Decomposer/Family/f__Pseudomonadaceae/seqs.align.html]

```
seq5            AGTGGGGAATATTGGACAATGGGCGAAAGCCTGATCCAGCCATGCCGCGTGTGTGAAGAA
seq57           AGTGGGGAATATTGGACAATGGGCGAAAGCCTGATCCAGCCATGCCGCGTGTGTGAAGAA
seq34           AGTGGGGAATATTGGACAATGGGCGAAAGCCTGATCCAGCCATGCCGCGTGTGTGAAGAA

seq5            GGTCTTCGGATTGTAAAGCACTTTAAGTTGGGAGGAAGGGttGTAgaTTAATACtcTGCa
seq57           GGTCTTCGGATTGTAAAGCACTTTAAGTTGGGAGGAAGGGCAGTAAGTTAATACCTTGCT
seq34           GGTCTTCGGATTGTAAAGCACTTTAAGTTGGGAGGAAGGGCAGTAAGcgAATACCTTGCT

seq5            aTTTTGACGTTACCGACAGAATAAGCACCGGCTAACTCTGTGCCAGCAGCCGCGGTAATA
seq57           GTTTTGACGTTACCaACAGAATAAGCACCGGCTAACTtcGTGCCAGCAGCCGCGGTAATA
seq34           GTTTTGACGTTACCGACAGAATAAGCACCGGCTAACTCTGTGCCAGCAGCCGCGGTAATA

seq5            CAGAGGGTGCAAGCGTTAATCGGAATTACTGGGCGTAAAGCGCGCGTAGGTGGTTTGTTA
seq57           CgaAGGGTGCAAGCGTTAATCGGAATTACTGGGCGTAAAGCGCGCGTAGGTGGTTcagcA
seq34           CAGAGGGTGCAAGCGTTAATCGGAATTACTGGGCGTAAAGCGCGCGTAGGTGGTTTGTTA

seq5            AGTTGGATGTGAAATCCCCGGGCTCAACCTGGGAACTGCATtCAAAACTGaCtgaCTAGA
seq57           AGTTGGATGTGAAATCCCCGGGCTCAACCTGGGAACTGCATCCAAAACTactgAGCTAGA
seq34           AGTTGaATGTGAAATCCCCGGGCTCAACCTGGGAACTGCATCCAAAACTGgCaAGCTAGA

seq5            GTAtGGTAGAGGGTGGTGGAATTTCCTGTGTAGCGGTGAAATGCGTAGATATAGGAAGGA
seq57           GTAcGGTAGAGGGTGGTGGAATTTCCTGTGTAGCGGTGAAATGCGTAGATATAGGAAGGA
seq34           GTAgGGcAGAGGGTGGTGGAATTTCCTGTGTAGCGGTGAAATGCGTAGATATAGGAAGGA

seq5            ACACCAGTGGCGAAGGCGACCACCTGGACTaATACTGACACTGAGGTGCGAAAGCGTGGG
seq57           ACACCAGTGGCGAAGGCGACCACCTGGACTgATACTGACACTGAGGTGCGAAAGCGTGGG
seq34           ACACCAGTGGCGAAGGCGACCACCTGGgCTcATACTGACACTGAGGTGCGAAAGCGTGGG

seq5            GAGCAAA
seq57           GAGCAAA
seq34           GAGCAAA
```
